# Preparation and Characterization of Semi-Alicyclic Polyimide Resins and the Derived Alignment Layers for Liquid Crystal Display Technology

**DOI:** 10.3390/polym12010217

**Published:** 2020-01-15

**Authors:** Hong-sheng Bi, Xin-xin Zhi, Peng-hui Wu, Yan Zhang, Lin Wu, Yao-yao Tan, Yan-Jiang Jia, Jin-gang Liu, Xiu-min Zhang

**Affiliations:** 1 Beijing Key Laboratory of Materials Utilization of Nonmetallic Minerals and Solid Wastes, National Laboratory of Mineral Materials, School of Materials Science and Technology, China University of Geosciences, Beijing 100083, China; 2103170020@cugb.edu.cn (H.-s.B.); 2003180031@cugb.edu.cn (X.-x.Z.); 1003182221@cugb.edu.cn (P.-h.W.); 2103170021@cugb.edu.cn (Y.Z.); 2003190023@cugb.edu.cn (L.W.); 2003190022@cugb.edu.cn (Y.-y.T.); 2003190024@cugb.edu.cn (Y.-J.J.); 2 School of Electrical Engineering, Beijing Jiaotong University, Beijing 100044, China

**Keywords:** semi-alicyclic polyimide, alignment layer, thin-film transistor-driven liquid crystal display device (TFT-LCD), voltage holding ratio, residual direct circuit voltage

## Abstract

Uniform alignment of rigid-rod liquid crystal (LC) molecules under applied voltage is critical for achievement of high-quality display for thin-film transistor-driven liquid crystal display devices (TFT-LCDs). The polymeric components that can induce the alignment of randomly aligned LC molecules are called alignment layers (ALs). In the current work, a series of organo-soluble polyimide (SPI) ALs were designed and prepared from an alicyclic dianhydride, hydrogenated 3,3′,4,4′-biphenyltetracarboxylic dianhydride (HBPDA), and various aromatic diamines, including 4,4′-methylenedianiline (MDA) for SPI-1, 4,4′-aminodianiline (NDA) for SPI-2, 3,3′,5,5′-tetramethyl-4,4′-diaminodiphenylmethane (TMMDA) for SPI-3, and 3,3′-diethyl-5,5′-dimethyl-4,4′-diaminodiphenylmethane (DMDEDA) for SPI-4. The derived SPI resins were all soluble in *N*-methyl-2-pyrrolidone (NMP). Four SPI alignment agents with the solid content of 6 wt.% were prepared by dissolving the SPI resins in the mixed solvent of NMP and butyl cellulose (BC) (NMP/BC = 80:20, weight ratio). Liquid crystal minicells were successfully fabricated using the developed SPI varnishes as the LC molecule alignment components. The SPI ALs showed good alignment ability for the LC molecules with the pretilt angles in the range of 1.58°–1.97°. The LC minicells exhibited good optoelectronic characteristics with voltage holding ratio (VHR) values higher than 96%. The good alignment ability of the SPI ALs is mainly attributed to the good comprehensive properties of the SPI layers, including high volume resistivity, high degree of imidization at the processing temperature (230 °C), good rubbing resistance, good thermal stability with glass transition temperatures (*T*_g_s) higher than 260 °C, and excellent optical transparency with the transmittance higher than 97% at the wavelength of 550 nm.

## 1. Introduction

Research and development (R&D) of advanced materials for display technology represents one of the most vigorous and active directions in material science and engineering areas [1]. The progress of functional materials greatly promoted the rapid development of advanced display technologies, such as liquid crystal display devices (LCDs), light-emitting diode (LED) display devices, organic LED (OLED) devices, and so on [2]. In the course of practicality and commercialization of LCD panels, the R&D of new materials plays an important role. For instance, the application of wide view (WV) films successfully solved the narrow angle problems that prohibited the practical application of LCD devices [3]. The application of high-performance alignment layers (ALs) for liquid crystal (LC) molecule alignment makes it possible to enrich the new modes of thin-film transistor-driven liquid crystal display (TFT-LCD) devices, such as in-plane switching (IPS) type, fringe field switching (FFS) type, vertical alignment (VA) type, and so on [4]. The R&D for high-performance ALs particularly attracted increasing attention in recent years due to their critical roles in affecting the display quality of TFT-LCDs, such as image-sticking issues, lightness, contrast, and so on [5].

Polyimides (PIs) are one of the most common ALs in TFT-LCDs due to their stable physical and chemical properties, which guarantee the good alignment ability for LC molecules and also good processing characteristics, such as good film-forming ability, excellent rubbing endurance, and so on [6,7,8,9,10]. Conventional wholly aromatic PI ALs are usually used with the form of the soluble precursor, poly(amic acid) (PAA) due to the insoluble nature for the cured aromatic PIs. In early LCD panel fabrication, such as the black and white mode of twisted nematic LCD (TN-LCD), PAA alignment agents are firstly coated on the indium tin oxide (ITO) glass substrates and then thermally cured at elevated temperatures as high as 300 °C to facilitate the imidization reaction. Then, the derived PI ALs are rubbed with specific rubbing cloth to induce the alignment of PI molecular chains [11,12,13]. The rubbing-induced anisotropy of the PI molecular chains along and perpendicular to the rubbing direction makes it possible to align the LC molecules [14]. Then, the LC components are injected into the sandwich cells composed of two pieces of ITO glass substrate coated with the rubbed PI ALs, thus forming the basic structure of the LCD panel.

In recent years, the property requirements for PI ALs greatly increased. For instance, for the current full-color IPS or FFS modes of TFT-LCDs, very severe properties are demanded for PI ALs, including a low curing temperature (≤230 °C) due to the wide use of temperature-sensitive components in the devices, a high voltage holding ratio (VHR) feature so as to endow the devices with high lightness and high contrast, and low residual direct circuit voltage (RDC) so as to prohibit the formation of image-sticking defects in the devices [15]. These requirements greatly promoted the R&D of novel PI ALs in past decades. Among the various methodologies adopted in the literature, the strategy of blending the PAA type and the preimidized soluble PI (SPI) type of alignment components seems to be the one of the most promising procedures to meet the property requirements mentioned above. Table 1 summarizes the structural characteristics of PAA and SPI components [16]. It can be clearly seen that the PAA type of alignment agent is characterized by a good adhesion to substrate, high rubbing endurance, and good RDC feature, while it is characterized by a poor storage stability, high curing temperature, and poor VHR feature due to the existence of polar carboxyl in the structures. However, these drawbacks could be remedied by a mixture of PAA type and the less polar SPI type agents. A combination of PAA and SPI components is expected to afford novel ALs with the best combined properties.

The optimal compositions of PI alignment agents mainly consist of resins (SPI + PAA), solvents, leveling agents, and additives [17]. PAA resin is usually prepared via the conventional polycondensation procedure of alicyclic dianhydride and aromatic diamine monomers [18,19]. The semi-alicyclic molecular structure is beneficial for prohibiting the intra- and intermolecular charge transfer in the PI molecular chains, thus endowing the AL with high voltage retention and good optical transmittance [20]. Compared with PAA resins, there are fewer varieties of SPI resins reported in the literature [21,22,23,24]. SPI resins usually possess semi-alicyclic molecular structures for the same reason mentioned above. However, the very limited types of alicyclic dianhydride monomers that can endow the PI resins with good solubility in organic solvents greatly hinder the R&D of high-performance SPI ALs.

In the current work, as part of our continuous research developing high-performance PI ALs for advanced TFT-LCDs [25,26], a series of novel semi-alicyclic SPI ALs were developed from the alicyclic dianhydride, hydrogenated 3,3′,4,4′-biphenyltetracarboxylic dianhydride (HBPDA), and various aromatic diamines. The effects of the molecular structures of the SPIs on their thermal, optical, and optoelectronic properties were investigated in detail.

## 2. Materials and Methods

### 2.1. Materials

Hydrogenated 3,3′,4,4′-biphenyltetracarboxylic dianhydride (HBPDA) was purchased from Weihai Newera Kesense New Materials Co. Ltd. (Shandong, China) and dried at 150 °C in vacuo for 24 h prior to use. The diamines, including 4,4′-methylenedianiline (MDA), 4,4′-aminodianiline (NDA), 3,3′,5,5′-tetramethyl-4,4′-diaminodiphenylmethane (TMMDA), and 3,3′-diethyl-5,5′-dimethyl-4,4′-diaminodiphenylmethane (DMDEDA), were all purchased from Tokyo Chemical Industry Co., Ltd. (Tokyo, Japan) and used as received. γ-Butyrolactone (GBL), *N*-methyl-2-pyrrolidone (NMP), *N,N*-dimethylacetamide (DMAc), butyl cellulose (BC), and other solvents were obtained from Sinopharm Chemical Reagent Co. Ltd. (Shanghai, China) and purified by distillation prior to use.

### 2.2. Characterization Methods

Absolute viscosity of the SPI alignment agents was measured using a Brookfield DV-II+ Pro viscometer (Brookfield Ametek, MA, USA) at 25 °C. Inherent viscosity of the SPI varnish was measured using an Ubbelohde viscometer (Mitong Electromechanical Tech. Co. Ltd., Shanghai, China) with a 0.5 g/dL NMP solution at 25 °C. The number average molecular weight (*Mn*) and weight average molecular weight (*Mw*) of the SPI resins were measured using a gel permeation chromatography (GPC) system (Shimadzu, Kyoto, Japan) with LC-20AD dual-plunger parallel-flow pumps (D1-LC), an SIL-20A total-volume injection-type auto-sampler, a CTO-20A column oven, and an RID-20A detector. HPLC-grade NMP was used as the mobile phase at a flow rate of 1.0 mL/min. Attenuated total reflectance Fourier-transform infrared (ATR-FTIR) spectra of the SPI ALs were recorded on an Iraffinity-1S FT-IR spectrometer (Shimadzu, Kyoto, Japan). Ultraviolet–visible light (UV–Vis) spectra of the SPI ALs were recorded on a Hitachi U-3210 spectrophotometer (Tokyo, Japan) at room temperature. Prior to testing, PI samples were dried at 100 °C for 1 h to remove the absorbed moisture. Wide-angle X-ray diffraction (XRD) was conducted on a Rigaku D/max-2500 X-ray diffractometer (Tokyo, Japan) with Cu-Kα1 radiation, operated at 40 kV and 200 mA. The chemical composition of SPIs was measured on a Flash EA 1112 elemental analyzer (Thermo Scientific, Waltham, MA, USA).

Thermogravimetric analysis (TGA) was performed on a TA-Q50 thermal analysis system (New Castle, DL, USA) at a heating rate of 20 °C/min in nitrogen. Differential scanning calorimetry (DSC) was recorded on a TA-Q100 thermal analysis system (New Castle, DL, USA) at a heating rate of 10 °C/min in nitrogen. Thermomechanical analysis (TMA) was recorded on a TA-Q400 thermal analysis system (New Castle, DE, USA) in nitrogen at a heating rate of 10 °C/min.

The volume resistivity (*ρ*_v_) of PI ALs was measured at room temperature according to the ASTM D-257-91 standard using a PC68 digital high-voltage and high-resistance megger instrument (Shanghai, China). The samples were dried at 120 °C for 1 h prior to measurement.

Solubility was investigated by mixing 1.0 g of the SPI resin and 9.0 g of the solvent tested (10 wt.% solid content), and then stirred for 24 h at room temperature. The solubility was determined visually as three grades: completely soluble (++), partially soluble (+), and insoluble (−), wherein completely soluble indicates a homogeneous and clean state without phase separation, precipitation, or gel formation, and insoluble indicates no change in the appearance of the resin.

### 2.3. PI Resin Synthesis and Varnish Preparation

The representative synthesis procedure for the SPI resins is presented as the preparation of SPI-1. Accurately weighted MDA (19.8260 g, 100 mmol) and newly distilled GBL (100.0 g) were added to a 500-mL three-necked, round-bottomed flask equipped with a mechanical stirrer, a Dean–Stark trap, and a nitrogen inlet. The mixture was stirred at room temperature for 10 min under the protection of nitrogen flow to afford a clear pale-yellow solution. Then, HBPDA (30.6310 g, 100 mmol) was added to the diamine solution through the adding funnel and was washed thoroughly with an additional volume of GBL (51.4 g). The molar ratio of the HBPDA dianhydride and the MDA diamine was 1:1, and the solid content of the reaction system was controlled to be 25 wt.%. Isoquinoline (0.5 g) was added as an imidization catalyst.After stirring in nitrogen for 1 h, toluene (150 mL) was then added as an azeotropic agent. The reaction mixture was heated to 180 °C and maintained for 6 h. During the reaction, the toluene–water azeotrope was distilled out of the system and collected in the Dean–Stark trap. After cooling to room temperature, the viscous solution was carefully poured into an excess of methanol to yield a silky white resin. The obtained SPI-1 resin was collected and dried at 80 °C in vacuo for 24 h.

The other PI resins, including SPI-2 (HBPDA/NDA), SPI-3 (HBPDA/TMMDA), and SPI-4 (HBPDA/DMDEDA), were prepared according to a similar procedure as mentioned above except that MDA was replaced by NDA for SPI-2, TMMDA for SPI-3, and DMDEDA for SPI-4.

SPI-1. Yield: 45.5 g (97%). Proton nuclear magnetic resonance (^1^H-NMR) (dimethyl sulfoxide (DMSO)-*d*_6_, ppm): 7.34–7.31 (*d*, 4H), 7.21–7.18 (*d*, 4H), 4.01 (*s*, 2H), 3.19–3.16 (*m*, 2H), 2.94–2.82 (*m*, 2H), 2.18–1.97 (*m*, 4H), 1.61–1.55 (*m*, 4H), 1.26–1.11 (*m*, 4H), and 0.96–0.90 (*m*, 2H). Elemental analysis: calculated for (C_29_H_28_N_2_O_4_)*_n_*: C, 74.34%; H, 6.02%, N, 5.98%. Found: C, 73.98%; H, 6.31%, N, 6.04%.

SPI-2. Yield: 45.1 g (96%). ^1^H-NMR (DMSO-*d*_6_, ppm): 8.62 (*s*, 1H), 7.26–7.21 (*m*, 8H), 3.18–3.16 (*m*, 2H), 2.95–2.93 (*m*, 2H), 2.21–1.97 (*m*, 4H), 1.60–1.58 (*m*, 4H), 1.28–1.12 (*m*, 4H), and 0.99–0.96 (*m*, 2H). Elemental analysis: calculated for (C_28_H_27_N_3_O_4_)*_n_*: C, 71.62%; H, 5.80%, N, 8.95%. Found: C, 71.33%; H, 6.02%, N, 9.03%.

SPI-3. Yield: 50.4 g (96%). ^1^H-NMR (DMSO-*d*_6_, ppm): 7.13–7.11 (*d*, 4H), 3.92 (*s*, 2H), 3.26–3.23 (*m*, 2H), 3.07–3.04 (*m*, 2H), 2.35–2.33 (*m*, 4H), 2.17–2.11 (*m*, 4H), 2.01(*m*, 12H), 1.66–1.64 (*m*, 4H), 1.28–1.12 (*m*, 4H), and 0.98–0.92 (*m*, 2H). Elemental analysis: calculated for (C_33_H_36_N_2_O_4_)*_n_*: C, 75.55%; H, 6.92%, N, 5.34%. Found: C, 75.17%; H, 7.05%, N, 5.66%.

SPI-4. Yield: 53.1 g (96%). ^1^H-NMR (DMSO-*d*_6_, ppm): 7.11–7.10 (*m*, 2H), 7.08–7.06 (*m*, 2H), 3.89 (*s*, 2H), 3.28–3.26 (*m*, 2H), 3.11–3.07 (*m*, 2H), 2.32–2.30 (*m*, 4H), 2.15–2.09 (*m*, 4H), 1.99 (*m*, 10H), 1.65–1.63 (*m*, 4H), 1.36–1.34 (*m*, 2H), and 1.04–0.97 (*m*, 6H). Elemental analysis: calculated for (C_35_H_40_N_2_O_4_)*_n_*: C, 76.06%; H, 7.29%, N, 5.07%. Found: C, 75.88%; H, 7.37%, N, 5.22%.

The fully dried SPI-1 resin was dissolved in the mixed solvent consisting of the newly distilled NMP and BC (NMP/BC = 80:20, weight ratio) at room temperature with the solid content of 6 wt.% to afford the SPI-1 varnish. Then, the varnish was continuously filtered through a 0.25-μm and 0.10-μm Teflon filter to remove the undissolved impurities. The absolute viscosity of the obtained varnish was measured. Then, the SPI-1 solution was spin-coated on a clean indium tin oxide (ITO) substrate. The thickness of the SPI-1 film for various measurements was controlled by regulating the spinning rate. SPI-1 films with different thicknesses were obtained by thermally baking the SPI-1 solution in flowing nitrogen according to the following heating procedure: 100 °C/1 h, 150 °C/1 h, 180 °C/1 h, 200 °C/1 h, and 230 °C/0.5 h.

### 2.4. Fabrication of Liquid Crystal (LC) Minicells

Indium tin oxide (ITO) glass substrates (size: 100 mm × 100 mm × 0.5 mm) were firstly cleaned with APP (Atmosphere Pressure Plasma, SE-15 K-A/K 1203-06, Dawonsys Co., Ltd., Gyeonggi-do, Korea) treatment and then wiped with deionized water and acetone and dried. The PI alignment agent was spin-coated on the ITO substrates (5 s from 0 to 1000 rpm; maintained for 5 s; 10 s from 1000 to 4000 rpm; maintained for 20 s; then stopped), followed by pre-baking at 90 °C for 90 s on a hot plate (model: HOT PLATE HI-400A, AS ONE Co., Ltd., Osaka, Japan) to give a uniform coating with the thickness of 85 ± 10 nm by adjustment of the spinning speed. Then, the PI-coated ITO substrates were main-cured for at 230 °C for 30 min to complete the imidization. The thickness of the PI ALs was measured using a Dektak XT profilometer (Bruker, Karlsruhe, Germany).

The PI-coated ITO substrates were treated with a semi-automatic rubbing machine (model: HY6018A, Qingdatianda Co., Ltd., Beijing, China). Rubbing strength (RS) is defined as RS = NM (2πrn/v − 1), where *N* is the cumulative rubbing number (*N* = 1 in the current work), *M* is the depth of fiber deformation of the rubbing cloth (*M* = 0.3 mm in the current work), *n* is the rotating speed of the roller per minute (*r* = 1000 rpm in the current work), *r* is the radius of the friction roller (*r* = 64.5 mm in the current work), and v is the moving speed of the stage (*v* = 30 mm/s). Thus, RS = 67.2 mm in the current work. Then, the rubbing-treated PI-ITO substrates were placed on the dispenser (model: 300DS, Musashi Co., Ltd., Tokyo, Japan), and the epoxy sealant was coated under vacuum. The substrates were preheated at 90 °C for 90 s on a hot plate. Finally, two ITO substrates coated with PI layers with the opposite rubbing direction were assembled face to face into a sandwich structure and placed in the oven at 120 °C for 10 min to solidify the sealant. The sandwich structure of the ITO substrates was cut into small minicells (10 mm × 10 mm × 0.5 mm) with a cutting machine (model: HXD500, Fenghua Co., Ltd., Shanxi, China) along the sealing frame. Then, the LC minicells were filled with positive Δε liquid crystals by means of a syringe and sealed with UV sealant. Finally, the LC minicells were placed in an oven at 120 °C for 1 h to finish the curing of the UV sealant.

The optoelectronic properties of the LC minicells, including pretilt angles (TPA) of LC molecules, voltage holding ratio (VHR), and residual directive circuit voltage (RDC), were measured. VHR and RDC were determined using an optoelectronic system (model: 6254, TOYO Co., Ltd., Tokyo, Japan). For the VHR measurement, the applied voltage was ±5V, the pulse width was 60 μs, and the frame period was 16.67 ms. For the RDC measurement, the direct current (DC) stress was 5 V, the discharge was 1 s, and the measurement was 2400 s. Pre-tilt angle (TPA) measurement of LC minicells was performed by the crystal rotation method using a liquid crystal property evaluation device (model: RETS-4600, Otsuka Electronics Co., Ltd., Osaka, Japan).

## 3. Results and Discussion

### 3.1. PI Synthesis and Alignment Agent Preparation

**Figure 1:**
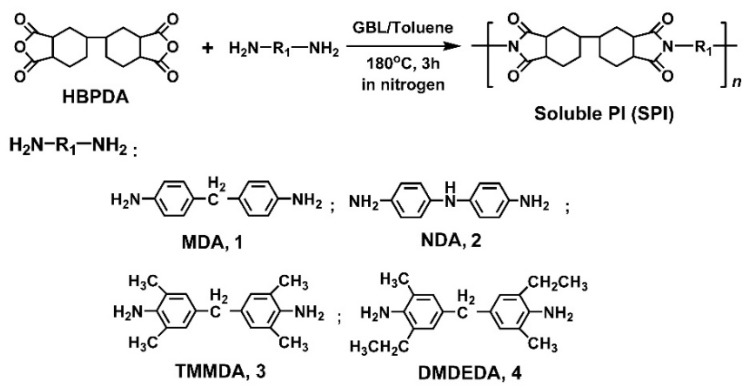
Preparation of soluble polyimide (SPI) resins.

A series of PI resins with enhanced solubility in organic solvents were designed and synthesized with a one-step high-temperature polycondensation procedure shown in Figure 1. For this target, the alicyclic dianhydride HBPDA with non-conjugated molecular structure was polymerized with several aromatic diamines with different structural characteristics. MDA is usually the standard diamine component for the preparation of PI ALs used in IPS or FFS-type TFT-LCDs due to the good alignment ability for LC molecules, good rubbing endurance due to the high surface hardness of the derived PI ALs, and good VHR feature due to the high electrical resistivity of the derived PI ALs [27]. TMMDA and DMDEDA diamines are all the alkyl-substituted derivatives of MDA, which, on one hand, possess the good property advantages of the MDA diamine, and, on the other hand, might improve the solubility of the derived PI resins. In addition, the pendent alkyl substituents in the TMMDA-PIs and DMDEDA-PIs might affect the alignment behaviors of the LC molecules [13]. NDA diamine contains a secondary amine (–NH–) linkage in the molecular structure. Since the lone-pair electrons are present on the nitrogen atom in NDA, they are easily attracted toward the electron-deficient carbonyl groups in the dianhydride moiety, leading to a charge transfer in the molecular chains of the NDA-PIs. As we know, the charge transfer (CT) theory was well used to explain a number of properties for PIs, such as colors, photodegradation, fluorescence, photoconductivity, electroconductivity, glass transition, and so on [28]. In the current work, the CT process was enhanced for SPI-2 due to the presence of lone-pair electrons on the nitrogen atom in NDA compared to that of other SPIs. The conjugated molecular chains facilitated the charge transfer in SPI-2. The charges in the diamine fragment are more easily attracted toward the electron-deficient carbonyl groups in the dianhydride moiety. This is beneficial for decreasing the charge accumulation in the PI. This feature endows the NDA-PI ALs with valuable properties whereby they could transfer the accumulated charges caused by the rubbing procedure so as to provide low RDC characteristics for the fabricated TFT-LCD panels [25,26]. However, the VHR features of the devices might be deteriorated by the use of NDA-PI ALs.

Four PI resins were prepared smoothly without gelling or precipitation occurring during the polycondensation. The PI resins with inherent viscosities (*η*_inh_) around 0.73–1.06 dL/g and number average molecular weights (*M*_n_s) in the range of 38,683–63,285 g/mol were obtained, as shown in Table 2. Basically, the alkyl-substituted SPI-3 and SPI-4 resins showed lower *η*_inh_ and *M*_n_ values compared to those of the SPI-1, which might be due to the relatively lower reactivity of the amino groups in TMMDA for SPI-3 and DMDEDA for SPI-4 caused by the steric effects of the *ortho*-substituted alkyl groups. SPI-2 possessed the highest *M*_n_ value, indicating the higher reactivity of the NDA diamine. Meanwhile, a higher polydispersity index (PDI) value was also observed for SPI-2. This might be attributed to the possible side reactions of the anhydride groups with the imino groups (–NH–), which increased the molecular weight distribution of the PI resin.

The solubility of the PI resins in various solvents was quantitatively evaluated, and the results are tabulated in Table 2. As expected, the current semi-alicyclic PI resins exhibited good solubility in all of the conventional good solvents for PIs, such as NMP, DMAc, and GBL. On the contrary, they were stable in the common leveling agents for PI varnishes, including butyl cellosolve (BC) and dipropylene glycol monomethyl ether (DPM). As mentioned before, the PI alignment agents usually consist of resins in both good solvents and leveling agents in order to achieve the uniform and smooth alignment layers. The dissolving behaviors of the current PI resins make them very suitable to be used as the alignment agents for TFT-LCDs.

**Figure 2:**
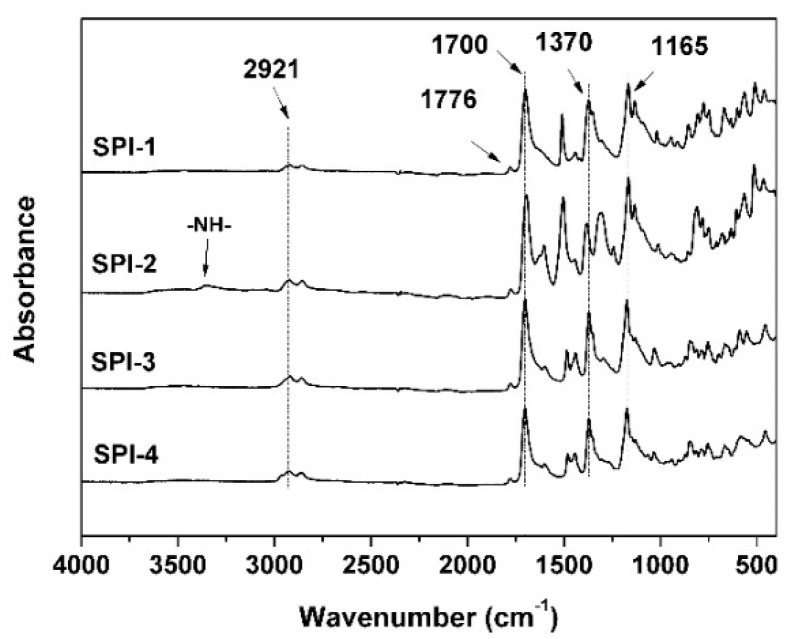
Attenuated total reflectance Fourier-transform infrared (ATR-FTIR) spectra of the polyimide (PI) resins.

The chemical structures of the developed PI resins were detected by infrared (IR) and nuclear magnetic resonance (NMR) measurements. Figure 2 shows the ATR-FTIR spectra of the PIs, together with the assignment of the characteristic absorptions of the polymers. The common characteristic absorptions in the PIs, including the imide units, saturated C–H (–CH– and –CH_2_–) in the HBPDA units, and the C=C stretching vibrations of benzene rings in the diamine units were all clearly recorded. For instance, the absorptions at 1776 cm^−1^ assigned to the asymmetrical carbonyl stretching vibrations, the peaks at 1700 cm^−1^ assigned to the symmetrical carbonyl stretching vibrations, and the peaks at 1370 cm^−1^ for the C–N stretching vibrations all confirmed the successful formation of imide rings. The absorptions at 2921 cm^−1^ and 2858 cm^−1^ assigned to the asymmetrical and symmetrical –CH_2_– stretching vibrations, respectively, identified the existence of cyclohexane units in the HBPDA moiety and the alkyl substituents in SPI-1, SPI-3, and SPI-4. For SPI-2, the absorption of –NH– was also detected at the wavenumber of 3350 cm^−1^, which originated from the incorporated NDA unit instead of the incomplete imidization of amide (–CONH–) groups in PAA. At last, peaks at 1165 cm^−1^ corresponded to the in-plane deformation of benzene ring.

**Figure 3:**
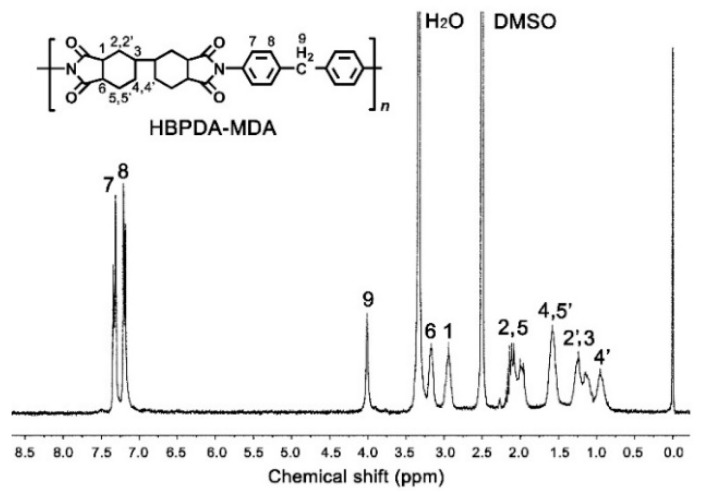
Proton nuclear magnetic resonance (^1^H-NMR) spectrum of SPI-1 resin.

The representative ^1^H-NMR spectrum of the SPI-1 resin is shown in Figure 3. It can be clearly seen that the spectrum was divided into two parts: the downfield of aromatic protons and the upfield of aliphatic protons. The protons located on the phenyl ring (H_7_, H_8_) in the diamine unit revealed the absorptions at the farthest downfield (chemical shift: 7.5–7.0 ppm) in the spectrum. The absorptions of the protons in the methylene (H_9_) appeared at the second farthest downfield in the spectrum. The protons *ortho*-substituted to the electron-withdrawing carbonyl group in the anhydride units, i.e., the H_1_ and H_6_ protons, showed relatively higher chemical shift values compared to the other protons in the cyclohexane rings. The proton pairs in the cyclohexane rings, including H_2,2′_, H_4,4′_, and H_5,5′_, displayed the different absorptions in the spectrum due to the different chemical environments of the protons.

In summary, the ATR-FTIR and ^1^H-NMR assignments were all in good agreement with the anticipated structure of the SPI-1 resin.

**Figure 4:**
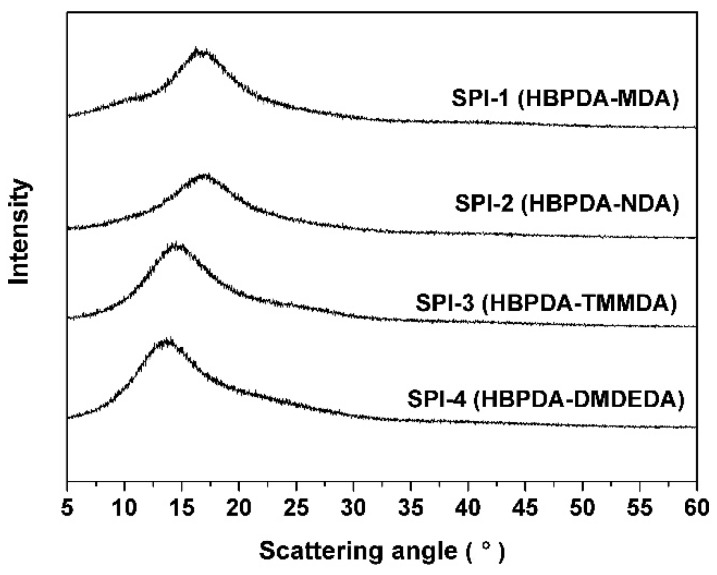
X-ray diffraction (XRD) spectra of the PI alignment layers (ALs).

The XRD spectra of the PI ALs as a function of Bragg scattering angle (2θ) are shown in Figure 4. All PI films exhibited an amorphous nature due to the loose molecular packing caused by the cyclohexane rings in the PIs. The average *d*-spacing was calculated based on Bragg’s law as follows: *n*λ = 2*d*sinθ, where *n* stands for an integer (*n* = 1), λ is the X-ray wavelength (λ = 1.54 Å), θ is the Bragg angle in degrees, and *d* is the *d*-spacing. The XRD spectra of the PI films showed diffraction peaks at 16.66° for SPI-1, 17.04° for SPI-2, 14.40° for SPI-3, and 13.52° for SPI-4. Correspondingly, the *d*-spacing or the inter-chain distance of the PIs was 5.31 Å for SPI-1, 5.20 Å for SPI-2, 6.14 Å for SPI-3, and 6.54 Å for SPI-4. This result corresponds well with the chemical structures of the PIs, among which SPI-4 derived from HBPDA and DMDEDA had the smallest molecular chain packing density due to the lateral methyl and ethyl substituents. On the contrary, SPI-1 and SPI-2 derived from the diamines without any substituent possessed relatively compact molecular chain packing, thus exhibiting smaller *d*-spacing values.

### 3.2. Thermal Properties

Good thermal and dimensional stability at elevated temperatures for PI ALs can usually provide stable LC alignments and, thus, they are critical for the practical applications of PI ALs. As mentioned before, most of the current PI ALs are semi-alicyclic PIs, whose thermal stability is usually inferior to that of wholly aromatic PIs. Thus, in the molecular design of advanced PI ALs for TFT-LCDs, thermal stable groups or structures are usually favorable. In the current research, the thermal stabilities of the PI ALs were investigated by TGA, DSC, and TMA measurements. The thermal data are summarized in Table 3.

**Figure 5:**
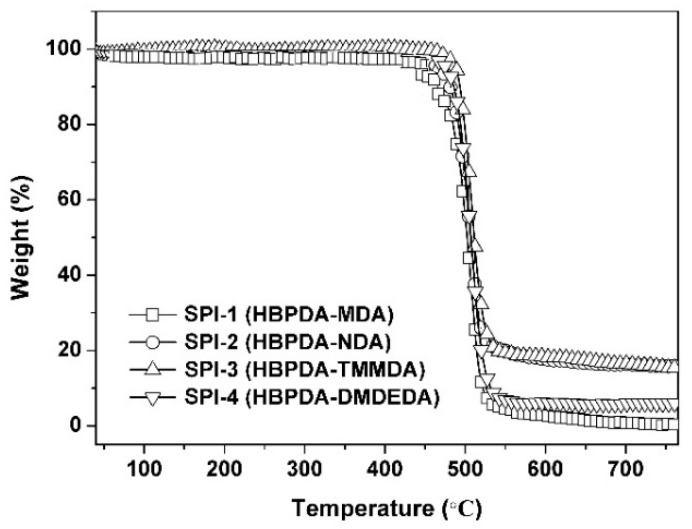
Thermogravimetric analysis (TGA) curves of the PI ALs.

Figure 5 presents the TGA curves of the PI ALs in the temperature range of 50 to 760 °C in nitrogen. All the designed and developed PIs exhibited good thermal stability up to 400 °C. The 5% weight loss temperature (*T*_5%_) of the PI ALs was in the range of 446.7–476.1 °C. After 450 °C, the PIs decomposed rapidly, leaving only 0.8–16.5 wt.% of their original weights at 700 °C. SPI-2 and SPI-3 showed somewhat higher thermal stability in the PI series in terms of the *R*_w700_ values.

**Figure 6:**
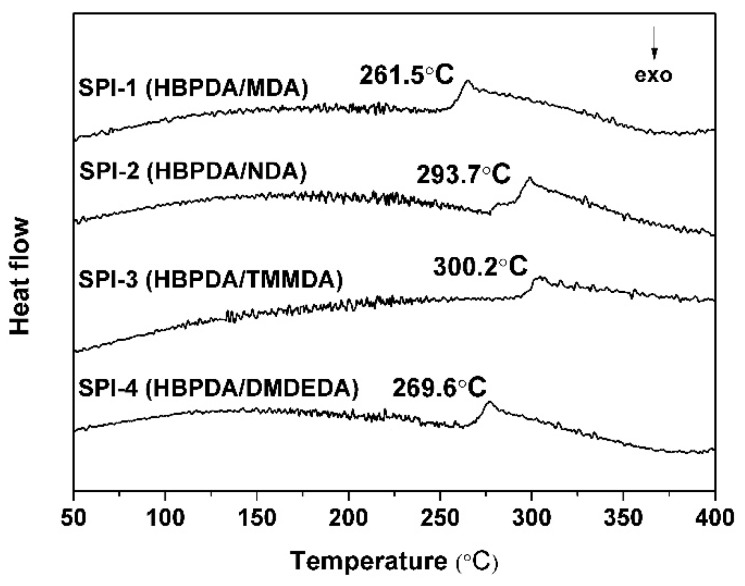
Differential scanning calorimetry (DSC) curves of the PI ALs.

The glass transition temperatures (*T*_g_) of the PI ALs were determined by the DSC measurements, as illustrated in Figure 6. All the PI ALs possessed good thermal stability with *T*_g_ values higher than 260 °C. As anticipated, SPI-3 with the *ortho*-substituted methyl groups exhibited the highest *T*_g_ value, while SPI-1 with the flexible methylene linkage showed the lowest one. The higher *T*_g_ for SPI-3 was mainly due to the substitution of methyl groups in the *ortho*-position to the imide ring, which restricted the rotation of the nitrogen atom along the phenyl ring [29]. This result increased the *T*_g_ of the polymer. Although the *ortho*-substituted alkyl groups also existed in SPI-4, the flexible ethyl substituents decreased the thermal stability of the polymer, resulting in a much lower *T*_g_ value. For SPI-1, there was a considerable decrease in the rigidity and internal rotation energy of the polymer chains due to the presence of the flexible methylene units, thus reducing the *T*_g_ value of the polymer. It is noteworthy that the SPI-2 exhibited the second highest *T*_g_ value in the series. This might be attributed to the slight crosslinking in the polymer chains due to the potential reaction between the secondary amine (–NH–) in the NDA moiety and the dianhydride units during polycondensation.

**Figure 7:**
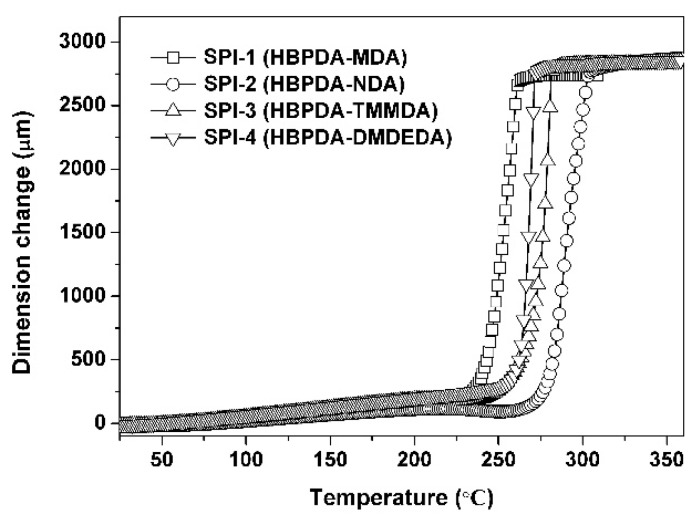
Thermomechanical analysis (TMA) curves of the PI ALs.

The high-temperature dimensional stability of the PI ALs was investigated by TMA measurements, and the results are shown in Figure 7, while the CTE values of the PI ALs are listed in Table 3. From the TMA plots, on one hand, we can deduce the thermal stability of the PI ALs in terms of the dimensional change during the heating. Interestingly, the TMA revealed different glass transition behaviors from the DSC measurements for SPI-2 and SPI-3. In the TMA measurements, SPI-2 exhibited a higher *T*_g_ value than that of SPI-3, which is opposite from the DSC measurements shown in Figure 6. This phenomenon could be attributed to the different sensitivity of these two polymers to the DSC and TMA measurements, and similar results were also reported in the literature [30]. On the other hand, the CTE of the PI samples was determined from the TMA measurements. In the temperature range of 50–200 °C, the PI ALs showed CTE values in the range of (53.2–81.6) × 10^−6^/K at a thickness of 25 μm, which is higher than that of wholly aromatic PI films, such as the poly(pyromellitic dianhydride-4,4′-oxydianiline) (PMDA-ODA) film (CTE: ~32 × 10^−6^/K) [31] and the indium tin oxide substrate (CTE: ~7.2 × 10^−6^/K) [32]. Nevertheless, the current PI ALs might exhibit good dimensional stability in the course of TFT-LCD panel fabrication due to the very thin thickness of several hundreds of angstroms in practical applications.

In summary, the current PI ALs possess good thermal stability which could be expected to guarantee stable alignment for the LC molecules.

### 3.3. Optical Properties

**Figure 8:**
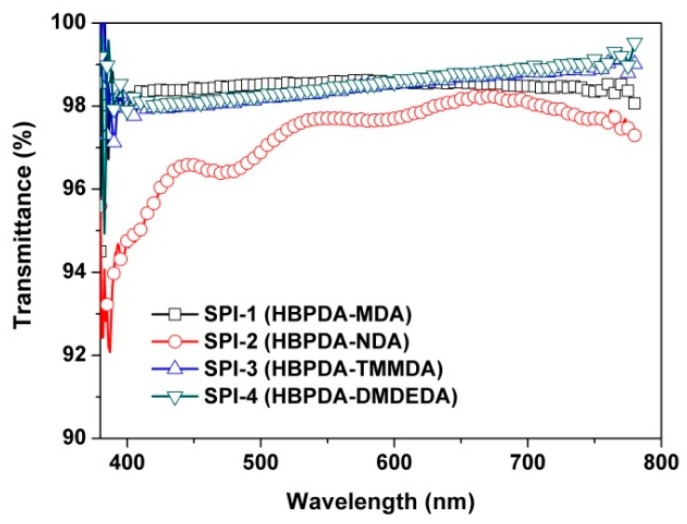
Ultraviolet–visible light (UV–Vis) spectra of PI ALs (thickness: ~85 nm).

Good optical transparency is usually critical for the practical applications of PI ALs due to the intrinsic low transmittance of TFT-LCD panels [33]. In order to improve the light efficiency of the backlight in TFT-LCDs, the optical transmittances of the layer components are required to be as high as possible. Figure 8 depicts the UV–Vis spectra of the PI ALs at the processing thickness around 85 nm, and the optical transmittance values at the wavelength of 550 nm (*T*_550nm_) for the PI ALs are tabulated in Table 4. All the PI ALs showed good optical transparency in the ultraviolet–visible light region with *T*_550_ values higher than 97.0%. SPI-2 showed the lowest *T*_550_ value due to the thermo-sensitive secondary amine (–NH–) linkage in the polymer chains, which was subject to oxidization during the hard baking process at elevated temperatures (230 °C for 30 min) even in the inert nitrogen atmosphere.

**Figure 9:**
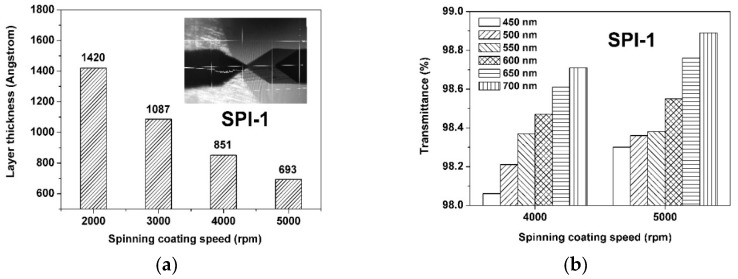
Influence of spinning coating speed on the physical parameters of SPI-1 alignment layer: (**a**) thickness (insert: thickness measurement by profilometer); (**b**) optical transmittance.

The thickness of the PI ALs was controlled at 85 nm by regulating the solution properties (solid contents and viscosities) and spinning coating parameters of the PI alignment agents, which is illustrated by the adjustment of SPI-1 as shown in Figure 9a. It can be clearly seen that the thickness of the SPI-1 AL decreased from 1420 Å at the spinning coating speed of 2000 rpm to 693 Å at the spinning coating speed of 5000 rpm. In the current work, the thickness of the PI ALs was adjusted to around 85 nm based on our previous work [26]. Thus, a spinning coating speed of 4000 rpm was used. At this coating condition, the SPI-1 AL showed increasing optical transmittance values in the range of 98.0%–98.7% when the wavelength increased from 450 nm to 700 nm, as shown in Figure 9b. The excellent optical transparency of the PI AL was mainly due to the less conjugated semi-alicyclic molecular structures of the polymers, which efficiently decreased the formation of intra- and intermolecular charge transfer complexes (CTCs) [34].

### 3.4. Optoelectronic Properties

**Figure 10:**
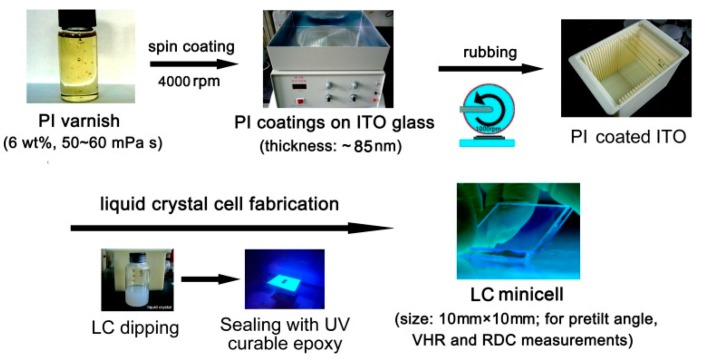
Fabrication of liquid crystal (LC) minicells with the developed PIs as the alignment layers.

In order to investigate the alignment effects of the developed PI ALs for LC molecules, the LC minicells were fabricated according to the procedure shown in Figure 10. The optoelectronic features of the minicells containing the currently developed PI ALs were investigated, and the results are listed in Table 4. The PI ALs showed good alignment abilities to the LC molecules with the pretilt angles (*θ*_p_) in the range of 1.58°–1.97°. These *θ*_p_ values are lower than that of standard wholly aromatic PI ALs, such as poly(pyromellitic dianhydride-4,4′-oxydianiline) (PMDA-ODA) (*θ*_p_: ~3°) [35]. This low-*θ*_p_ feature is highly desired for the IPS or FFS modes of TFT-LCD due to the reduced light leakage for the panels [36]. For the current work, a lower pretilt angle resulted in a better contrast ratio for the devices. It is well established that PI ALs containing alicyclic segments usually provide lower *θ*_p_ values compared to wholly aromatic analogues [37]. This feature was attributed to the decreasing polarity of the semi-alicyclic PI ALs, resulting in a reduced interaction between the PI ALs and the LC molecules.

At last, the effects of chemical structures of the PI ALs on the VHR and RDC features of the LC minicells were investigated. As compared in Table 1, the SPI ALs usually provided LCD devices with higher VHR and higher RDC features, while the PAA ALs showed lower VHR and low RDC characteristics. High VHR and low RDC features are highly desired for TFT-LCD devices. The former is highly related to the lightness and contrast parameters, and the latter is related to the image-sticking features of the TFT-LCDs. For practical applications, the VHR should be as high as possible and the RDC as low as possible in order to achieve a high-quality display. The property of the PI ALs is one of the most important factors affecting the VHR and RDC features of the fabricated LC panels. High purity, low ion density, low molecular polarity, high surface and volume resistivity, and low charge migration of PI ALs are highly desirable for improving the VHR feature. However, high ion density, high molecular polarity, and effective charge transfer pathway are usually demanded for PI ALs in order to achieve a low RDC feature. In practical applications, these two mutually contradictory parameters (VHR and RDC) should be considered simultaneously. It was reported in the literature that the current density of the PI film from PAA was higher than that of the PI film from an SPI in the same electric field [38]. Thus, a blend of SPI and PAA seems to be one of the most effective procedures to achieve this goal.

**Figure 11:**
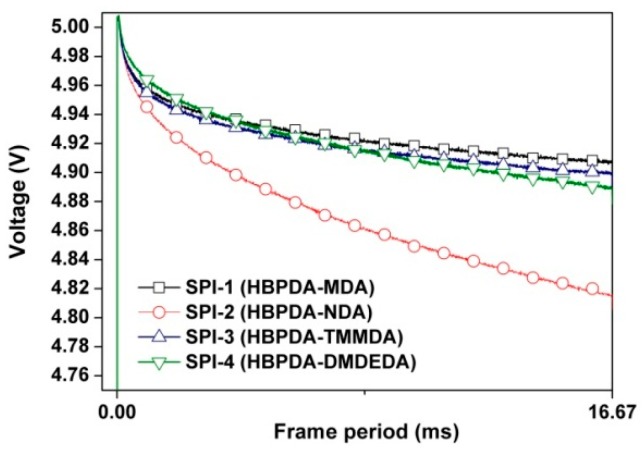
Voltage holding ratio (VHR) plots of the LC cells fabricated with the PI ALs.

**Figure 12:**
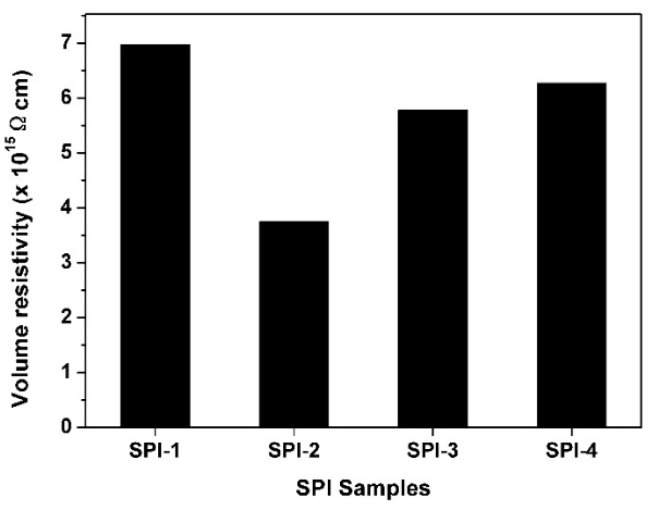
Volume resistivity of PI ALs.

In the current work, the VHR feature plots of the LC minicells are shown in Figure 11. All the PI ALs exhibited high VHR values over 97% except SPI-2. The lone pair of electrons on the N atom in the NDA moiety was easily attracted by the electron-deficient carbonyl groups in the imide rings, leading to a charge transfer in the molecular chains of SPI-2. This structural characteristic resulted in the decrease of VHR for SPI-2. Meanwhile, this low VHR feature for SPI-2 was consistent with the electrical resistivity (*ρ*_v_) values of the PI ALs, as shown in Figure 12. Generally, PI films are known for their high volume resistivity. However, introduction of the NDA component slightly decreased the *ρ*_v_ values of the polymer. The PI ALs showed decreased *ρ*_v_ values in the order of SPI-1 (6.97 × 10^15^ Ω∙cm) > SPI-4 (6.28 × 10^15^ Ω∙cm) > SPI-3 (5.78 × 10^15^ Ω∙cm) > SPI-2 (3.71 × 10^15^ Ω∙cm). Although the relatively lower *ρ*_v_ value for SPI-2 was not beneficial for obtaining high VHR feature for the fabricated LC minicells, it was quite desirable for achieving a good RDC feature.

**Figure 13:**
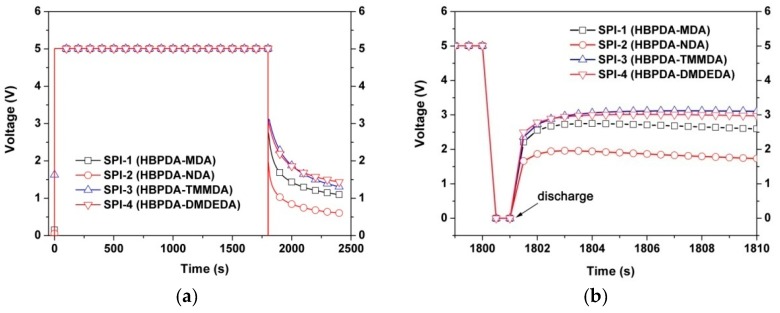
Residual direct current voltage (RDC) plots of the LC cells fabricated with the PI ALs: (**a**) whole period; (**b**) period immediately after discharging.

Figure 13 presents the RDC plots of the LC minicells fabricated with the newly developed PI ALs. In the RDC measurements, when the direct circuit (DC) voltage of 5 V was applied, the charge accumulated on the surface of PI ALs. After the applied voltage was switched off (Figure 13b), the LC molecules were exposed to a residual voltage. The decay behaviors of this residual voltage were recorded, as shown in Figure 13a. It can be seen that the residual charges remained for a longer period of time for SPI-1, SPI-3, and SPI-4 minicells than for the SPI-2 minicell. That is to say, for the former devices, the image might be retained by the residual charges for a long time even after the next voltage is applied. This phenomenon of a sticking image is highly undesirable in the fabrication of TFT-LCD panels. Comparatively, SPI-2 exhibited a better charge release capacity than its counterparts. This was mainly due to the existence of charge migration pathway in the molecular structure of SPI-2. The applied voltage of 5 V was rapidly released by SPI-2, and a relatively low RDC value of 605 mV was left at the time of 2400 s.

## 4. Conclusions

As one of the important components for PI alignment agents, SPI plays very important roles in maintaining the high VHR feature of TFT-LCD devices. Various SPI resins based on alicyclic dianhydride, HBPDA, and aromatic diamines were developed in the current work. In summary, the derived semi-alicyclic PI ALs exhibited good comprehensive properties, including low curing temperature, high thermal stability, and good optical transmittance. The fabricated LC minicells showed good optoelectronic performance, including low pretilt angles for LC molecules and high VHR features. The high RDC feature of the SPI ALs is expected and could be remedied by mixing with PAA components. The corresponding work is under investigation in our laboratory and will be reported in the near future.

## Figures and Tables

**Table 1:** Structural characteristics of poly(amic acid) (PAA) and soluble polyimide (SPI) components and the effects on the liquid crystal display (LCD) devices. VHR—voltage holding ratio; RDC—residual direct circuit voltage.

Items	PAA Type	SPI Type
Storage stability	Poor	Good
Printability	Good	Moderate
Curing temperature	Moderate to high	Low
Adhesion to substrate	High	Moderate
Rubbing endurance	High	Moderate to poor
Volume resistivity	Low	High
VHR feature of devices	Poor	High
RDC feature of devices	High	Poor

**Table 2:** Inherent viscosities, molecular weights, and solubility of SPI resins.

PI	*η*_inh_^a^ (dL/g)	Molecular Weight ^b^			Solubility ^c^				
		*M*_n_ (g/mol)	*M*_w_ (g/mol)	PDI	NMP	DMAc	GBL	BC	DPM
SPI-1	1.06	55,407	104,198	1.88	++	++	++	−	−
SPI-2	0.88	63,285	127,154	2.01	++	++	++	−	−
SPI-3	0.86	38,683	72,209	1.87	++	++	++	−	−
SPI-4	0.73	43,857	80,054	1.83	++	++	++	−	−

^a^ Inherent viscosities measured with a 0.5 g/dL PI solution in NMP at 25 °C; ^b^ *M*_n_: number average molecular weight; *M*_w_: weight average molecular weight; PDI: polydispersity index, PDI = *M*_w_/*M*_n_; ^c^ ++: Soluble; +: partially soluble; −: insoluble. NMP: *N*-methyl-2-pyrrolidone; GBL: γ-butyrolactone; BC: butyl cellosolve; DPM: dipropylene glycol monomethyl ether.

**Table 3:** Thermal properties of polyimide (PI) alignment layers (ALs).

PI	*T*_g_ (°C) ^a^	*T*_5%_ (°C) ^b^	*T*_10%_ (°C) ^b^	*R*_w700_ (%) ^c^	CTE (×10^−6^/K) ^d^
SPI-1	261.5	461.8	476.8	0.8	62.2
SPI-2	281.7	468.3	481.5	16.4	53.2
SPI-3	300.2	446.7	454.2	16.5	79.0
SPI-4	269.6	476.1	486.0	5.9	81.6

^a^*T*_g_: glass transition temperature; ^b^*T*_5%_, *T*_10%_: temperatures at 5% and 10% weight loss, respectively; ^c^*R*_w700_: residual weight ratio at 700 °C in nitrogen; ^d^ CTE: linear coefficient of thermal expansion in the range of 50–200 °C.

**Table 4:** Optical properties of the PI ALs and the optoelectronic features of the liquid crystal (LC) cells.

PI	*d*^a^ (Å)	*T*_550_^b^ (%)	*θ*_p_^c^ (^o^)	VHR ^d^ (%)	RDC ^e^ (mV)
SPI-1	851	98.4	1.58	97.81	1098
SPI-2	850	97.7	1.62	96.11	605
SPI-3	855	98.3	1.92	97.58	1312
SPI-4	853	98.2	1.97	97.57	1438

^a^*d*: thickness of PI alignment film; ^b^*T*_550_: transmittance at the wavelength of 550 nm; ^c^*θ*_p_: pretilt angles of LC molecules; ^d^ VHR: voltage holding ratio; ^e^ RDC: residual direct current voltage.
